# Aero-TiO_2_ three-dimensional nanoarchitecture for photocatalytic degradation of tetracycline

**DOI:** 10.1038/s41598-024-82574-6

**Published:** 2024-12-28

**Authors:** Vladimir Ciobanu, Tatiana Galatonova, Tudor Braniste, Pavel Urbanek, Sebastian Lehmann, Barbora Hanulikova, Kornelius Nielsch, Ivo Kuritka, Vladimir Sedlarik, Ion Tiginyanu

**Affiliations:** 1 Centre of Advanced Research in Bionanoconjugates and Biopolymers, “Petru Poni” Institute of Macromolecular Chemistry, 41A, Grigore Ghica Voda Alley, 700487 Iasi, Romania; 2 National Centre for Materials Study and Testing, Technical University of Moldova, 168, Stefan Cel Mare Av, 2004 Chisinau, Moldova; 3 Centre of Polymer Systems, Tomas Bata University in Zlin, 5678, tr. Tomase Bati , CZ 76001 Zlin, Czech Republic; 4 Institute for Metallic Materials (IMW), Leibniz Institute of Solid State and Materials Research (IFW Dresden), 20, Helmholtzstrasse , 01069 Dresden, Germany

**Keywords:** TiO_2_, Aeromaterials, Tetrapod, Tetracycline, Photocatalysis, Materials science, Nanoscience and technology, Chemical physics

## Abstract

One of the biggest issues of wide bandgap semiconductor use in photocatalytic wastewater treatment is the reusability of the material and avoiding the contamination of water with the material itself. In this paper, we report on a novel TiO_2_ aeromaterial (aero-TiO_2_) consisting of hollow microtetrapods with Zn_2_Ti_3_O_8_ inclusions. Atomic layer deposition has been used to obtain particles of unique shape allowing them to interlock thereby protecting the photocatalyst from erosion and damage when incorporated in active filters. The performance of the aero-TiO_2_ material was investigated regarding photocatalytic degradation of tetracycline under UV and visible light irradiation. Upon irradiation with a 3.4 mW/cm^2^ UV source, the tetracycline concentration decreases by about 90% during 150 min, while upon irradiation with a Solar Simulator (87.5 mW/cm^2^) the concentration of antibiotic decreases by about 75% during 180 min. The experiments conducted under liquid flow conditions over a photocatalyst fixed in a testing cell have demonstrated the proper reusability of the material.

## Introduction

Pharmaceutical industries contribute significantly to water pollution through the discharge of various chemical compounds, including antibiotics^1,2^. Tetracycline, a widely used antibiotic in both human and veterinary medicine, is among the pharmaceutical compounds frequently detected in aquatic environments, especially in highly populated areas. Conventional wastewater treatment methods often fail to completely remove these compounds, leading to their accumulation in the environment and potential ecological disruptions^3^. Therefore, there is an urgent need for efficient and sustainable strategies to diminish antibiotic pollution in wastewaters.

Various nanomaterials have been explored for photocatalytic degradation of antibiotics. These include but are not limited to zinc oxide (ZnO)^4^ and other metal oxides^5,6^, graphene-based materials^7^, and doped semiconductors^8^. Each nanomaterial exhibits unique properties and photocatalytic mechanisms, offering a diverse toolkit for addressing antibiotic pollution in wastewaters.

Among the various nanomaterials, titanium dioxide (TiO_2_) has attracted significant attention for its exceptional photocatalytic properties, which enable the degradation of organic pollutants under UV or visible light irradiation^9^. The unique structural and physico-chemical properties of TiO_2_ make it an ideal candidate for photocatalytic applications, including wastewater treatment^10^.

The widespread use of TiO_2_ for a variety of applications including photocatalysis can lead to increased effects on organisms and the environment. However, despite the enormous efforts undertaken in recent years to determine the real risk, the results achieved do not speak clearly and the technological advantages of using TiO_2_ outweigh^11^.

While TiO_2_-based nanomaterials have demonstrated promising results in photocatalytic degradation of antibiotics, several challenges remain to be addressed. Doping or functionalization with various elements^12^ or creating a mixture phase between TiO_2_ and other compounds^13–15^ could influence the photocatalysis process. Variations in the physicochemical properties of TiO_2_ nanoparticles, such as crystalline phase, particle size, and surface morphology, can significantly influence their photocatalytic performance. TiO_2_ can be fabricated at the nanoscale in various shapes and crystalline structures by different techniques^16^, each of them having advantages and disadvantages. TiO_2_ nanotubes fabricated by simple electrochemical etching of Ti foils were successfully used for the degradation of dyes such as Methylene Blue or Rhodamine B^17,18^. The tubular shape at the nanoscale can also add other functionalities to its photocatalytic properties. This shape enables its use as nanoengines activated by UV external light, making them suitable for applications such as water purification, drug delivery systems and others^19,20^.

In this paper, we report on a novel nanomaterial composed of TiO_2_ hollow microtetrapods fabricated by Atomic Layer Deposition (ALD) technique using a sacrificial ZnO template. While TiO_2_-based nanomaterials are extensively used for photocatalytic degradation, the hollow microtetrapod design provides a unique morphology not previously explored in the context of antibiotic degradation. The high surface area and tubular geometry enhance contact with pollutants, while the interconnected structure is advantageous in dynamic systems, as it provides mechanical stability. Furthermore, the material’s reusability in continuous flow reactors addresses a major challenge in scaling photocatalytic systems for industrial applications. Through these innovations, this work contributes with a durable and efficient approach for reducing antibiotic contamination in wastewater, combining material stability with effective photocatalytic degradation under visible and UV light irradiation. The importance of aero-TiO_2_ as a photocatalytic material resides also in its great potential to be used in self-propelled microengines^20,21^, which will open the way to novel applications in microfluidics.

## Results and discussion

**Fig. 1:**
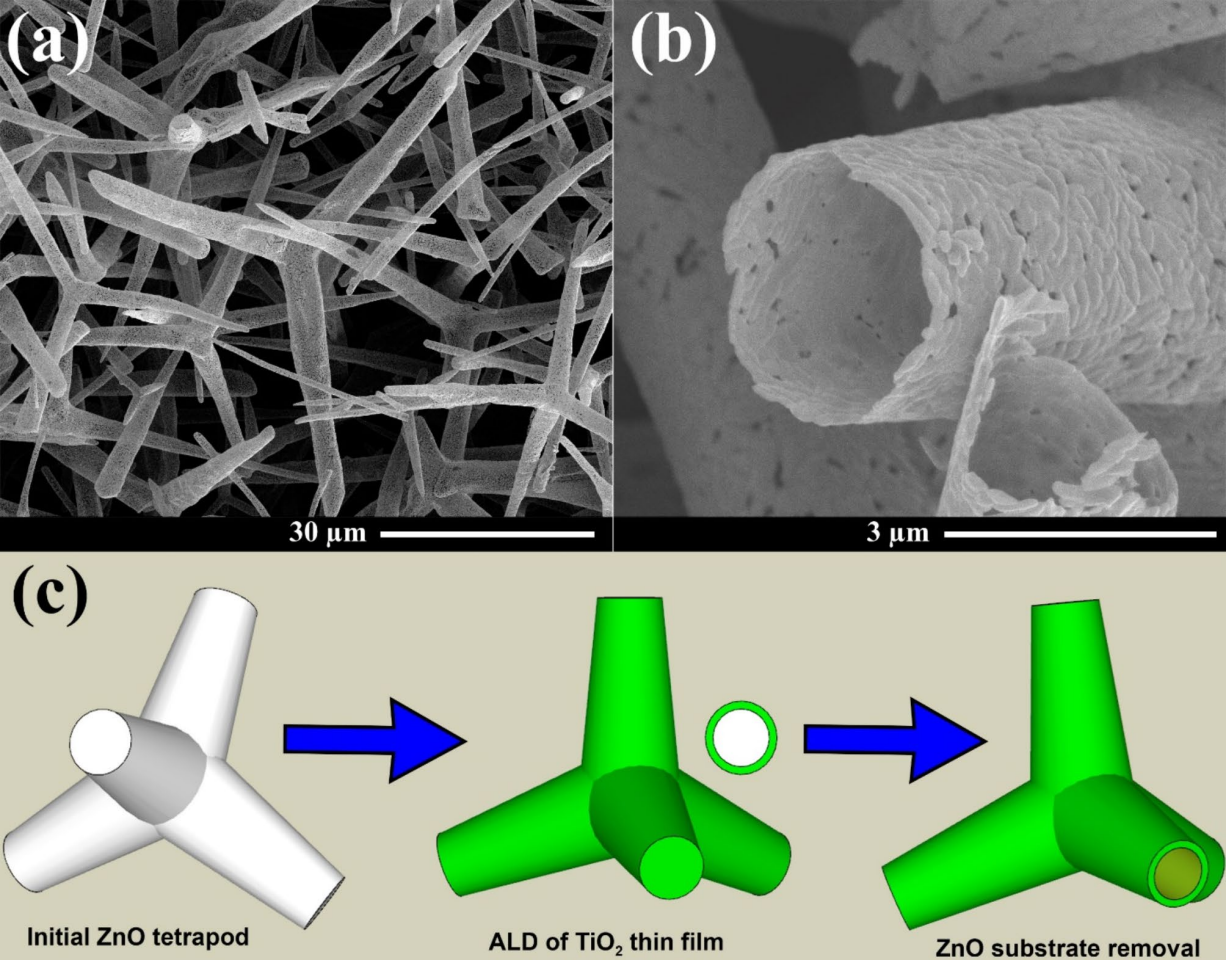
(**a, b**) SEM images of the aero-TiO_2_ material consisting of hollow microtetrapods, and (**c**) the schematic route of the aeromaterial preparation, starting with initial ZnO microtetrapods, atomic layer deposition of TiO_2_ on their surface (also illustrated in cross section) and, finally, removal of sacrificial ZnO substrate in the presence of HCl and H_2_ gases at 800 °C.

The morphology of the hollow TiO_2_ microtetrapods is illustrated in Fig. 1. The dimensions of the arms of aero-TiO_2_ microtetrapods vary in the range from 20 to 40 µm in length and 1 to 3 µm in diameter. The wall thickness of the TiO_2_ microtubes is around 50 nm.

**Fig. 2:**
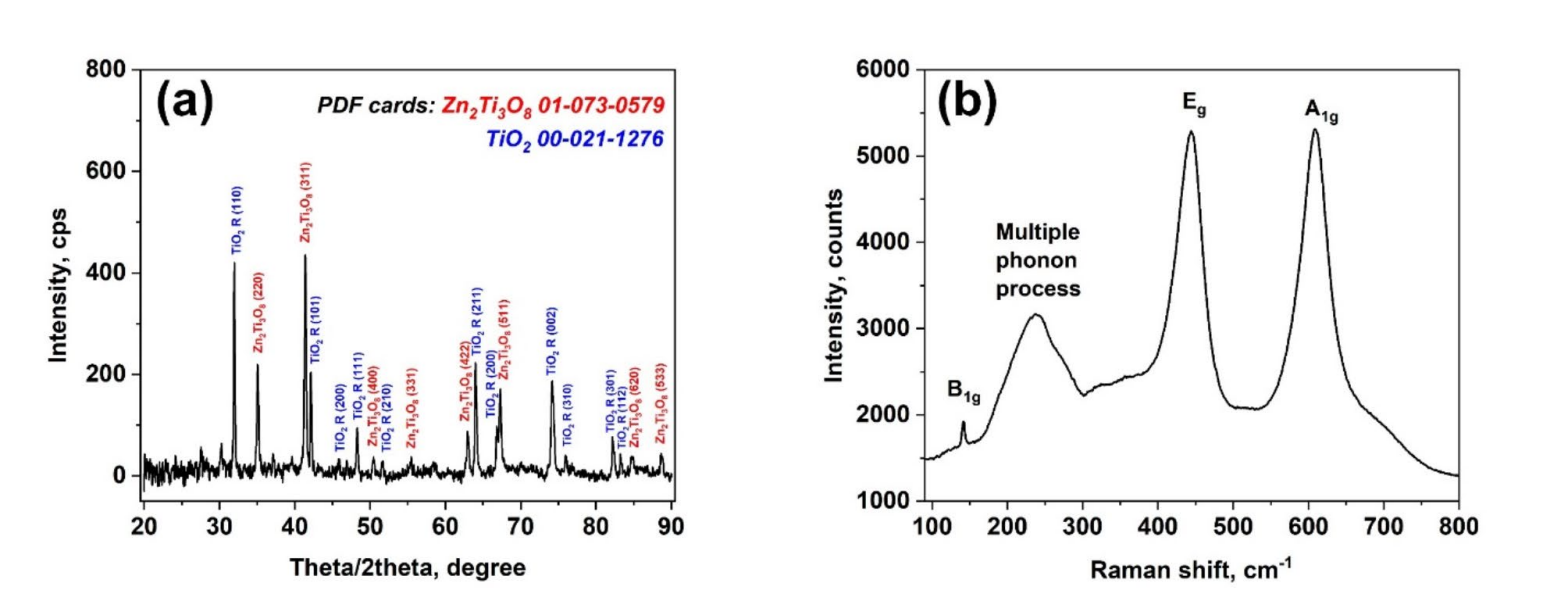
XRD pattern (**a**) and Raman spectrum (**b**) of the fabricated aero-TiO_2_ material.

The XRD pattern shown in Fig. 2a demonstrates the presence of the rutile phase TiO_2_ (JCPDS 00–021-1276) and the ternary compound Zn_2_Ti_3_O_8_ (JCPDS 01–073-0579). All diffraction lines of the TiO_2_ and Zn_2_Ti_3_O_8_ were indexed by tetragonal TiO_2_ with the space group P42/mnm(136) and cubic Zn_2_Ti_3_O_8_ with the space group Fd-3 m(227).

The sizes of crystallites were determined considering the main peaks of the compounds and were found to be 50 nm for rutile TiO_2_ and 36 nm for Zn_2_Ti_3_O_8_, which were determined by the Scherrer equation (Eq. 1):

1 $$D=0.89\lambda /\beta \text{ cos}\theta$$

where, *λ* is the wavelength (Co Kα, *λ* = 1.7903 Å), *β* is the full width at the half-maximum (FWHM) and *θ* is the diffraction angle.

It was found previously that aero-TiO_2_ can be obtained in a mixture of anatase–rutile compound with Zn_2_TiO_4_ inclusions by using the same ALD process with subsequent selective wet chemical etching of ZnO sacrificial template^13^. Higher annealing temperature and the different approach we used for the ZnO removal allowed one to obtain a composite consisting of rutile phase TiO_2_ and cubic Zn_2_Ti_3_O_8_.

The XRD data are corroborated by the Raman scattering analysis (Fig. 2b). The rutile structure of TiO_2_ belongs to the space group $D\genfrac{}{}{0pt}{}{14}{4h}$ and it has four Raman active vibrations: A_1g_ + B_1g_ + B_2g_ + E_g_. The observed peaks at 447 cm^−1^ and 612 cm^−1^ are attributed to the E_g_, and A_1g_ modes, respectively. Second order scattering features can also be visible in the spectrum, the most intensive one being at 238 cm^−1^ ^22^.

By using the Kubelka–Munk equation (Eq. 2), the optical band gap of the sample was determined from the diffuse reflectance spectrum:

2 $${\left[\alpha h\nu \right]}^{p}=A\left(h\nu -Eg\right),$$

where *α* is the optical absorption coefficient, *hν* is the photon energy, *A* is a constant of proportionality, and exponent *p* is determined by the transition type of the material: *p* = 2 for direct allowed transitions, *p* = 2/3 for direct forbidden transition, *p* = 1/2 for indirect allowed transitions, and *p* = 1/3 for indirect forbidden transitions.

Since TiO_2_ rutile phase is known as a direct transition semiconductor, the function used for plotting was

3 $${\left[F\left(R\right)h\nu \right]}^{2}=\left[h\nu -Eg\right],$$

**Fig. 3:**
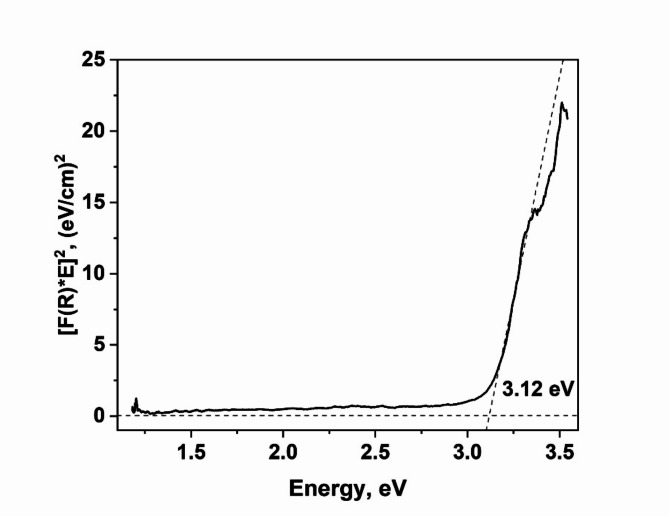
Optical bandgap determined from UV–visible diffuse reflectance spectrum.

The optical band gap energy of the material was determined by the extrapolation of the slope to *F(R)* → 0 from the plot *[F(R)·hν]*^2^ vs. *hν*, as shown in Fig. 3.

According to the modified Kubelka–Munk function, the UV–visible diffuse reflectance spectra show that TiO_2_ hollow microtetrapods have the bandgap of 3.12 eV. For the purpose of comparison one can note that anatase and rutile phases of TiO_2_ have a bandgap of around 3.0 eV and 3.2 eV, respectively^23^, while Zn_2_Ti_3_O_8_ has a calculated bandgap of 3.55 eV ^24^.

**Fig. 4:**
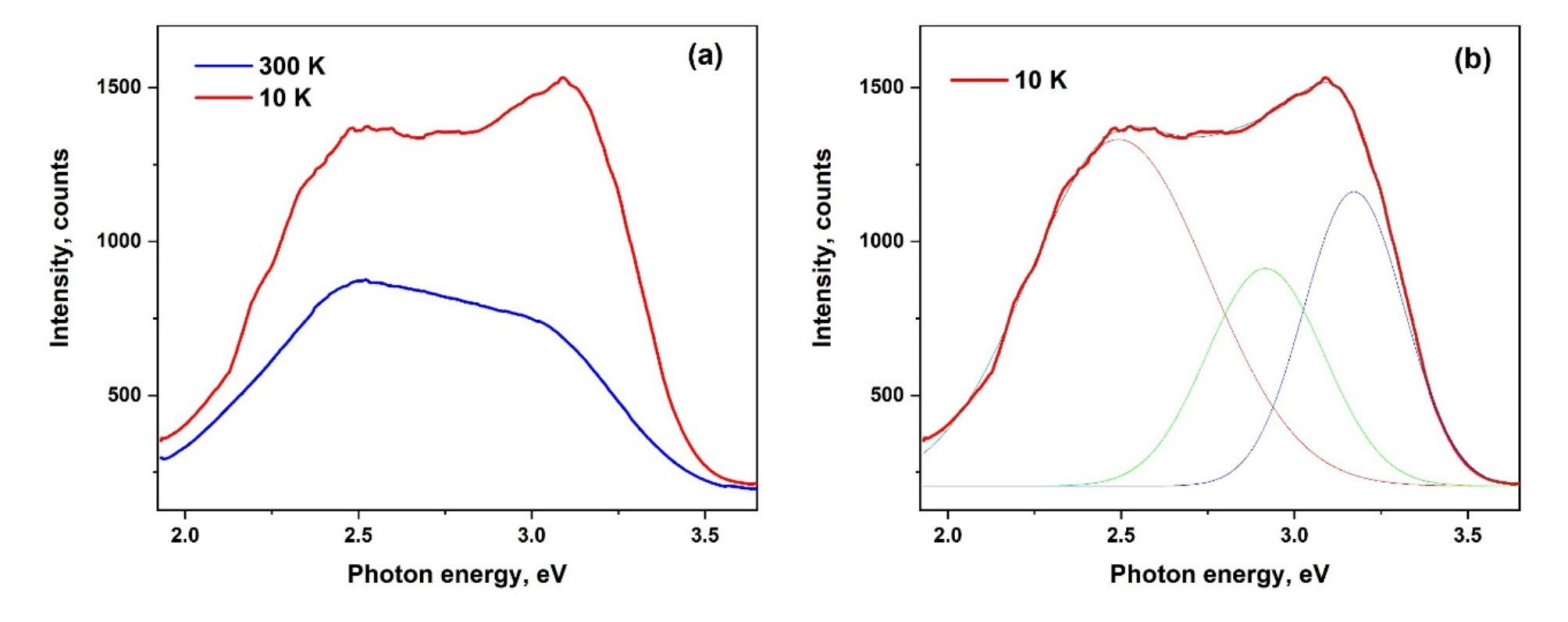
PL spectra of aero-TiO_2_ at 10 K and 300 K (**a**) and the deconvoluted spectrum of PL recorded at 10 K (**b**).

Photoluminescence (PL) measurements were performed to evaluate the presence of defects in the synthesized samples. The PL spectra from Fig. 4a span a broad energy range from 2.0 to 3.5 eV. Notably, as the temperature rises from 10 K to room temperature, the intensity of the high-energy band decreases more significantly than that of the low-energy band.

The deconvolution of the PL spectrum presented in Fig. 4b reveals that it comprises three distinct energy bands: one green band and two violet bands. The green band is centered at 2.5–2.6 eV, while the first and second violet bands are located respectively at 2.9–3.0 eV and 3.15 eV.

One can assign the high-energy emission band at 3.15 eV to near-bandgap transitions, while the violet PL band at 2.9 eV may be associated with an unidentified defect. The green band has previously been linked to the recombination of self-trapped excitons formed from carrier polarons^25^. It is suggested that oxygen vacancies facilitate effective trapping of carriers or polarons and, simultaneously, the efficient charge separation allows for electron and hole accumulation or trapping at distinct sites, potentially on the surface. Given the extensive surface areas of aeromaterials, one may expect surfaces to play a major role in their photoluminescence behavior.

The kinetics of the tetracycline photodegradation was fitted according to the pseudo-first-order model (Eq. 4):

4 $$\mathit{ln}\left(\frac{{C}_{0}}{{C}_{t}}\right)=kt,$$

where, *C*_0_ and *C*_*t*_ represent the concentrations of tetracycline in solutions at irradiation time *t* = 0 min and *t*, respectively, and *k* represents the degradation rate (min^-1^).

**Fig. 5:**
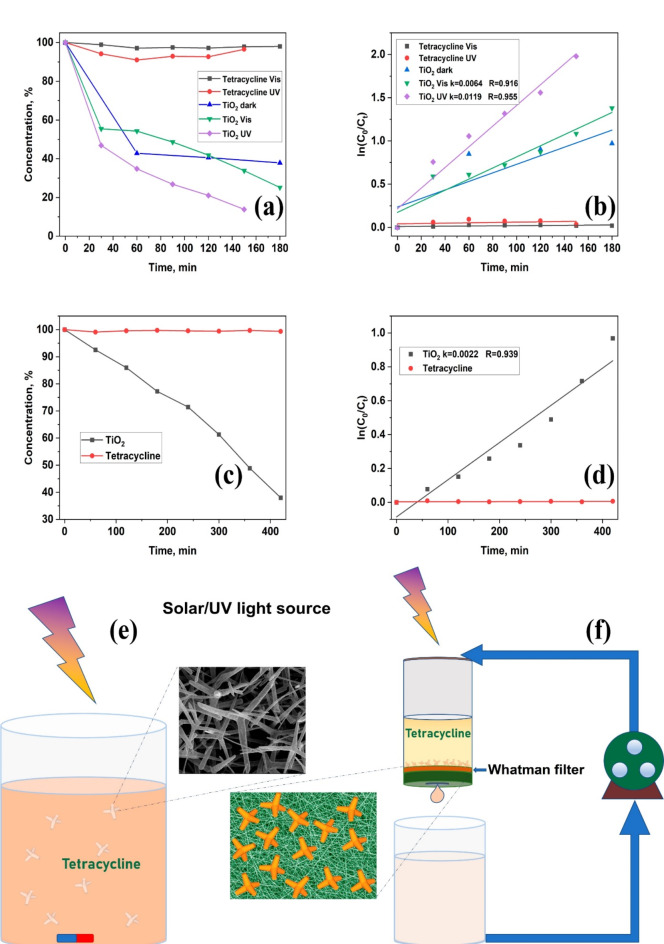
Photocatalysis performance of aero-TiO_2_ for the degradation of tetracycline under visible or UV (**a**) and under UV light irradiation in the continuous solution flow conditions (**c**) and their degradation rates (**b, d**); the schematics of the experimental setup for the photocatalysis tests (**e, f**).

Without the photocatalyst, the tetracycline concentration in the solution is not significantly influenced by irradiation with visible or UV light. When aero-TiO_2_ is added to the solution (Fig. 5e), the concentration of tetracycline decreases by about 75% when irradiated with visible light for 180 min. Upon irradiation with UV light, the photocatalysis process is faster, and the tetracycline concentration decreases by about 90% during 150 min (Fig. 5a). The degradation rates of tetracycline were estimated to be about 0.0064 min^-1^ and 0.0120 min^-1^ upon irradiation with visible and UV light, respectively, as shown in Figs. 5b.

Previously, Wu et al. have demonstrated that TiO_2_ P25 nanoparticles with the surface area of about 55 m^2^/g are able to degrade tetracycline with a ratio of about 0.038 min^-1^ under 350 nm irradiation, and the photocatalytic efficiency decreases with the increase of the wavelength irradiation source, down to 0.00055 min^-1^ at 850 nm ^26^. Despite the differences in testing conditions, these results still can be roughly compared with those from our work. The observed enhanced photocatalytic performance of aero-TiO_2_ can be attributed to the presence in the nanocomposite structure of Zn_2_Ti_3_O_8_ inclusions decreasing the rate of recombination of photogenerated electron–hole pairs, thus allowing them to reach the photocatalyst surface^27–29^.

Table 1 provides a summary of the existing knowledge on tetracycline photodegradation using various structures of TiO_2_ photocatalysts, emphasizing the performance of these materials and reaction parameters.

**Table 1:** Photocatalytic efficiency of different TiO_2_-based nanomaterials upon tetracycline degradation.

Material	Initial concentration of tetracycline	Nanoparticles concentration	Removal efficiency	Degradation time	References
Cu_2_O − TiO_2_ Nanotubes	30 mg/L	1.5 g/L	100%	180 min	^30^
TiO_2_-P25 nanoparticles	10 mg/L	0.2 g/L	25%	120 min	^26^
N-doped TiO_2_ nanoparticles	10 mg/L	0.2 g/L	66%	120 min	^31^
TiO_2_ nanoparticles on CuO sheets	50 mg/L	10 g/L	95%	90 min	^32^
Au − TiO_2_/PVDF nanocomposite	10 mg/L	30 g/L	75%	120 min	^33^
TiO_2_/natural pyrite	30 mg/L	2 g/L	100%	180 min	^34^
CQDs/TiO_2_	20 mg/L	0.2 g/L	62%	120 min	^35^
TiO_2_/p-BC	30 mg/L	0.1 g/L	94%	120 min	^36^
Aero-TiO_2_	10 mg/L	0.5 g/L	90%	150 min	This work

There are three main types of active species which mainly contribute to the photocatalytic degradation of tetracycline, namely ^·^O_2_^−^, ^·^OH and h^+^ as was previously observed by other authors^37^. ^·^O_2_^−^ and h^+^ species have a major role in the photocatalysis under UV and only ^·^O_2_^−^ becomes important under visible light irradiation^38^. The electrons from the valence band are excited to the conduction band, reacting with O_2,_ leading to the formation of ^·^O_2_^−^ species, which further react with the adsorbed tetracycline molecules at the material surface, while h^+^ species directly contribute to the oxidation of tetracycline^39^.

Previous studies have demonstrated that during photocatalytic oxidation reactions, oxygen vacancy defects in ZnO-TiO_2_ nanocomposite materials serve as active sites for capturing photoinduced electrons, significantly enhancing photocatalytic efficiency. Additionally, oxygen vacancies facilitate the adsorption of environmental oxygen onto the sample, leading to strong interactions between the photoexcited electrons captured by these vacancies and the adsorbed oxygen molecules^40^.

The ability to fabricate nanocomposites with precise control opens new pathways for bandgap engineering, enabling alignment of the conduction and valence bands of composite materials with the HOMO and LUMO molecular orbitals of organic compounds targeted for photocatalytic degradation. Under these conditions, photogenerated electrons transfer from the conduction band of one component to that of another, while photogenerated holes similarly move between valence bands. Additionally, transition of the excited electrons from organic molecules to the conduction bands of the nanocomposite components generate reactive species that drive chemical reactions^41^. Given the substantial specific surface area of the synthesized aeromaterials, it is also likely that surface states play a critical role in modulating the valence band edge, thereby enhancing photocatalytic properties under visible-light irradiation, including those relevant to water splitting.

In the experiment performed under continuous liquid flow conditions using UV light with a density of 3.2 mW/cm^2^ (see Fig. 5f), the concentration of tetracycline decreases by about 65% during seven hours of irradiation with a degradation rate of 0.0022 min^-1^ (Fig. 5c and d). After the full degradation of tetracycline, the material was repeatedly used in three more consecutive tests. It was observed that the degradation performance was not influenced, thus demonstrating the reusability of the material. Hence, the 3D shape of our material with 2D features, such as the wall thickness of about 50 nm, makes it suitable for incorporation in active filters for water treatment without the risk of water contamination with the active material.

## Materials and methods

### Synthesis of aero-TiO_2_

Aero-titania was obtained by growing thin layers of TiO_2_ using the ALD technique on a substrate consisting of sacrificial templates of ZnO microtetrapods. Flame Transport Synthesis approach was used to obtain ZnO microtetrapods^42^ which were kindly provided by Prof. Rainer Adelung from Kiel University, Germany. First, pellets with dimensions of 10 × 10 × 4 mm^3^ consisting of ZnO microtetrapods were fabricated in a steel mold under pressure, with a controlled density and porosity of the material. Then, TiO_2_ was deposited using a thermal ALD reactor (Veeco Savannah S200 from Veeco Instruments Inc., Plainview, New York, NY, USA), utilizing TiCl_4_ as Ti precursor and deionized water as oxygen source. The deposition process was performed at 150 °C. High purity nitrogen was used as carrier gas at a flow rate of 20 sccm. The pulse and purge times were 0.2/120/0.015/120 s (TiCl_4_/N_2_/H_2_O/N_2_) for a single ALD deposition cycle. The growth rate was determined to be about 0.16 nm/cycle and the final thickness of the deposited TiO_2_ was about 50 nm. Thickness measurements were performed on reference Si wafers located close to the sample using a spectroscopic ellipsometer (SENpro, SENTECH Instruments GmbH, Berlin, Germany). ZnO sacrificial material was etched in a Hydride Vapor Phase Epitaxy (HVPE) system using HCl gas and H_2_ at 800 °C.

### Materials characterization

The morphology of the materials was investigated by a scanning electron microscope Tescan TS5130 at 10 kV accelerating voltage. For the crystalline quality investigation, a Rigaku Miniflex-600 powder diffractometer equipped with a Co Kα source radiation (*λ* = 1.7903 Å) operating at 40 kV and an emission current of 15 mA was used. The measurements were performed from 20 to 90° at a scanning speed of 6°/min. The photocatalytic performance was investigated by the changes in the absorption spectra of the solution containing tetracycline by using a PerkinElmer 1050 UV/Vis spectrophotometer, from which the concentration in % was determined. The Raman spectra were recorded at room temperature using a Nicolet DXR system with an excitation laser beam of 532 nm. UV–Vis diffuse reflectance spectroscopy (DRUV-Vis) in the range from 265 to 800 nm was performed by a modular fiber spectrometer AvaSpec 2048–2 (Avantes, The Netherlands).

### Photocatalytic activity of aero-TiO_2_

The tetracycline used in the experiments was acquired from Sigma Aldrich (CAS #60548). Prior to mixing the aero-TiO_2_ in the solution, the material was thermally treated at 300 °C in air for one hour to change its surface properties from hydrophobic to hydrophilic. The photocatalytic investigations were performed under both UV and visible light in two different configurations. In the first case, 5 mg of aero-TiO_2_ was mixed with 10 ml of tetracycline solution with a concentration of 10 mg/L. The solution was continuously mixed with a stirrer at 250 rpm while being irradiated laterally with visible light from a Solar Simulator Pico Solar G2V using the standard Solar Spectrum AM1.5 g with a power density of 87.5 mW/cm^2^, or irradiated from the top with a Focused UV lamp type C-10A-HE with power density of 3.4 mW/cm^2^. 1 ml of solution was extracted every 30 min, centrifuged to avoid the presence of the material in the suspension and then the absorbance spectra were collected. In the second configuration, the tetracycline degradation was investigated under liquid flow conditions. For this, a testing cell was fabricated from an opened tube with Whatman filter at the bottom, which allowed solution flow in a second tank from which it was recirculated by a peristaltic pump with the set flow condition of 2.5 ml/min. 5 mg of material was initially mixed with 5 ml of water and poured on the Whatman filter, forming a layer of tetrapods after sedimentation or leading to impregnation into the filter in case of smaller tetrapods or broken arms of the tetrapods.

A UV lamp (*λ* = 365 nm), with power density of 3.2 mW/cm^2^ was used as an irradiation source (see Fig. 5e). A total amount of 50 ml of solution was used in the experiment. 1 ml solution was collected every 60 min from the underneath reservoir and the concentration of tetracycline was determined from the absorption spectra by investigating the intensity decrease of the peak at 270 nm. The absorption spectra were recorded in PMMA cuvettes.

## Conclusion

The novel aeromaterial fabricated by the ALD technique, composed of hollow microtetrapods of TiO_2_ with a wall thickness of 50 nm, proved to be efficient for photocatalytic degradation of tetracycline under UV or visible light irradiation. The UV/Vis spectroscopy confirmed a decrease in tetracycline concentration by about 90% under UV and about 75% under visible light irradiation within three hours, showing degradation rates of about 0.0120 min^-1^ and 0.0064 min^-1^, respectively. The fabricated material consists of TiO_2_ rutile phase with Zn_2_Ti_3_O_8_ inclusions according to XRD analysis and has an optical bandgap of 3.12 eV according to diffuse reflectance measurements. These features make it comparable with the performance of other TiO_2_ based nanostructures reported in the literature. However, the added value of this novel material is its micrometric size scale and morphological suitability for incorporating the hollow microtetrapods in textile supports, thus opening the possibility of using the material fixed on a solid substrate in a continuous flow photoreactor which imparts the reusability and excludes the contamination of water with TiO_2_ nanoparticles.

## Data Availability

The corresponding author (V.C.) can provide the datasets presented in this study upon reasonable request.
